# Transition-Based Constrained DFT for the Robust and Reliable Treatment of Excitations in Supramolecular Systems

**DOI:** 10.1021/acs.jctc.1c00548

**Published:** 2022-04-26

**Authors:** Martina Stella, Kritam Thapa, Luigi Genovese, Laura E. Ratcliff

**Affiliations:** † Department of Materials, Imperial College London, London SW7 2AZ, U.K.; ‡ Université Grenoble Alpes, CEA, IRIG-MEM-L_Sim, Grenoble 38000, France; § The Abdus Salam International Centre for Theoretical Physics, Condensed Matter and Statistical Physics, Trieste 34151, Italy; ∥ Centre for Computational Chemistry, School of Chemistry, University of Bristol, Bristol BS8 1TS, U.K.

## Abstract

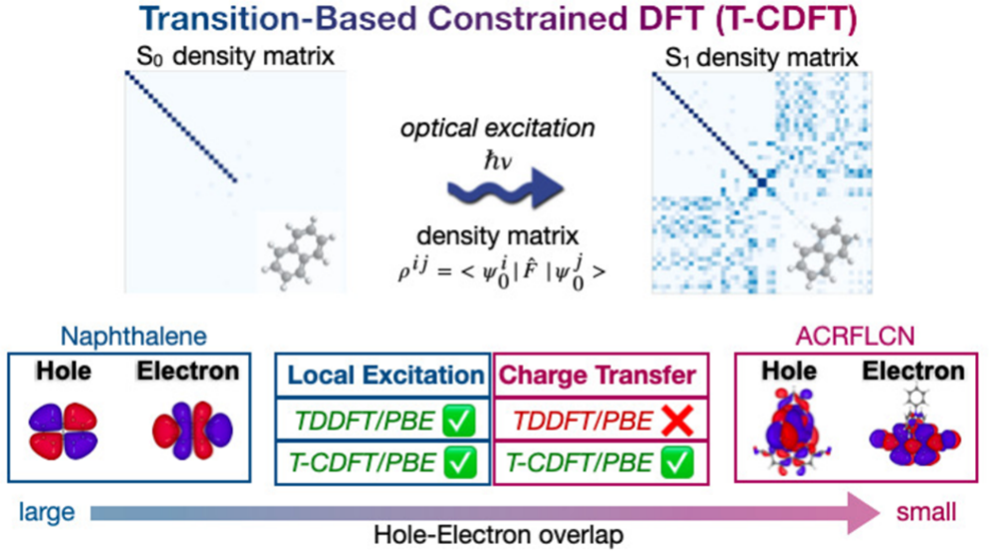

Despite the variety of available computational approaches, state-of-the-art methods for calculating excitation energies, such as time-dependent density functional theory (TDDFT), are computationally demanding and thus limited to moderate system sizes. Here, we introduce a new variation of constrained DFT (CDFT), wherein the constraint corresponds to a particular transition (T), or a combination of transitions, between occupied and virtual orbitals, rather than a region of the simulation space as in traditional CDFT. We compare T-CDFT with TDDFT and ΔSCF results for the low-lying excited states (S_1_ and T_1_) of a set of gas-phase acene molecules and OLED emitters and with reference results from the literature. At the PBE level of theory, T-CDFT outperforms ΔSCF for both classes of molecules, while also proving to be more robust. For the local excitations seen in the acenes, T-CDFT and TDDFT perform equally well. For the charge transfer (CT)-like excitations seen in the OLED molecules, T-CDFT also performs well, in contrast to the severe energy underestimation seen with TDDFT. In other words, T-CDFT is equally applicable to both local excitations and CT states, providing more reliable excitation energies at a much lower computational cost than TDDFT cost. T-CDFT is designed for large systems and has been implemented in the linear-scaling BigDFT code. It is therefore ideally suited for exploring the effects of explicit environments on excitation energies, paving the way for future simulations of excited states in complex realistic morphologies, such as those which occur in OLED materials.

## 1 Introduction

**Figure 1:**
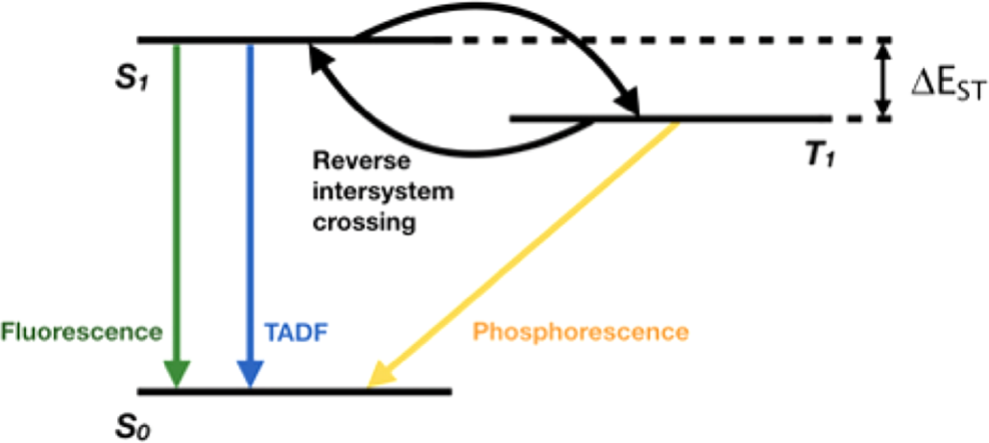
Simplified Jablonski diagram for TADF emitters. Reverse intersystem crossing, represented in the figure by the arrow going from T_1_ to S_1_ (with the intersystem crossing going from S_1_ to T_1_), is thermally activated when the energy difference between the two excited states is smaller or comparable to *k*_b_*T*.

Studying excited states in molecules and extended systems is one of the major ongoing challenges in physics, chemistry, and materials science due to the complexity of the underlying electronic structure. Nonetheless, an accurate characterization of excitation energies is crucial for a fundamental understanding of systems of technological interest, for example, solar cells,^1^ organic light emitting diodes,^2,3^ and chromophores in biological systems.^4^ One example of interest is thermally activated delayed fluorescence (TADF) emitters, which have gained a spotlight in recent years as a new type of organic light emitting diode (OLED).^2,3^ This is due to their promising maximum theoretical internal quantum efficiency (IQE) of 100%.^5−7^ TADF relies on a reverse intersystem crossing (RISC) mechanism (triplet-to-singlet energy up conversion, illustrated with a simplified Jablonski diagram in Figure 1) to achieve such high efficiency without employing expensive noble metal ions. TADF is however only possible at appreciable rates if the singlet–triplet splitting, Δ*E*_ST_, (defined in Figure 1), is smaller than or comparable to *k*_b_*T*, where *k*_b_ is the Boltzmann constant and *T* the temperature. Therefore, accurate prediction of Δ*E*_ST_ is a key but challenging element for designing more efficient TADF emitters. Excited states in TADF can be a mixture of charge transfer (CT) and local excitations (LEs), while their nature can vary with both chemical structures and changes in the molecular conformation.^8^

Experiments for investigating excitations can be challenging because of factors such as technical setups and short lifetimes. In addition, the classification and interpretation of the nature of different excitations (*i.e.*, valence state, CT, and LE) are often based on empirical data. *Ab initio* simulations therefore represent a valuable tool. However, as the example of TADF also highlights, there are many challenges that lie within the computational modeling of excited states, including the need to go beyond gas-phase simulations and instead consider realistic morphologies.^9^ In this context, it is necessary to develop a methodology which is able to reliably capture the excited electronic structure, while accounting for both conformational and environmental effects of the full system and still maintaining an affordable computational cost.

In this paper, we present a new computational approach motivated by the desire to simulate excitations in large systems for applications such as TADF. In order to motivate our approach, we first present an overview of currently available *ab initio* approaches for excited-state calculations, many of which have been developed in recent years as a result of community efforts to provide accuracy (see, *e.g.,* ref (10)) and precision benchmarks^11^ for molecular quantities beyond the ground state.

### 1.1 Density Functional Theory-Based Methods for Simulating Excited States

Density functional theory (DFT)^12,13^ has established itself as one of the most promising approaches for studying excitations in molecules and large systems, mainly due to its notoriously favorable trade-off between accuracy and computational cost. However, it is well known that, in its standard form, DFT falls short when describing excited states because of the ground-state nature of its formulation. For this reason, a range of different DFT-based methodologies have been developed in order to better account for excited electronic states.

ΔSCF (self-consistent field)^14^ is the simplest DFT-based approach for computing excitation energies. For a given excited state of interest, the energy splitting is defined as Δ*E*_SCF_^*n*^ = *E*_*n*_ – *E*_0_, where *E*_0_ is the ground-state energy and *E*_*n*_ is the energy of an “excited” state, labeled by *n*, which is obtained by manually controlling the occupation of the Kohn–Sham (KS) states as the system reaches self-consistency. The ΔSCF approach has been used with wide success because of its simplicity and low computational cost. It has, for a long time, been justified in cases where the excited state corresponds to the lowest state of a given symmetry,^15^ while its applicability has been also extended, such that it gets a formal justification in the general case.^16^

Linear-response (LR) time-dependent DFT (TDDFT)^17,18^ is the most commonly used method for investigating excitations in molecular systems as it often provides good agreement with experiments. Despite being well established and more affordable than sophisticated post-Hartree–Fock methods such as CCSD(T) and CASSCF,^19^ LR-TDDFT nonetheless has limitations which prevent it from being feasibly employed in modeling realistic morphologies, such as those in which TADF emitters are employed. First, its computational cost is still too onerous for modeling systems larger than a few hundred atoms,^20^ although various approaches have been developed for treating large systems, for example, linear scaling TDDFT^21^ and GPU-accelerated approaches^22^ and subspace-based approaches.^23^ Second, it notoriously fails when describing CT states with routinely used semi-local functionals.^24,25^ The latter issue in particular has been extensively studied, and a number of solutions are nowadays available, of which the most successful is the use of range-separated hybrid functionals.^26^ However, such functionals are still not widely available and can make calculations more expensive, while good performance often necessitates the tuning of the functional parameters for the system in question.^25^

Another DFT-based method for studying excited states is constrained DFT (CDFT).^27,28^ In CDFT, a constraint is added to the density, following which the energy is found by minimizing the density with this additional condition. In its most common form, a specific electronic charge is constrained to a region of simulation space. If opportunely guessed, such a constraint can correspond to a specific excited state where the electronic density is well localized within the region and takes into account, *by design*, the self-consistent response of the system to the imposition of such a constraint. For this reason, CDFT has performed very successfully for molecular systems with an obvious spatial separation between donor and acceptor regions.^28−35^ Such a simple approach naturally overcomes some of the well-known limitations of DFT, for example, the self-interaction problem and the resulting delocalization errors, making CDFT particularly appropriate for treating CT states and an asset for modeling exciton formation. CDFT is conceptually intuitive and follows the same scaling as DFT (generally scaling with the cube of system size). However, it is most appropriate where some information is known about the excitation in question (where and how much charge to impose). For a comprehensive review of CDFT, we refer the reader to ref (36).

The simplicity of its framework has also made CDFT attractive for the development of new variations. Recently, Ramos and Pavanello^37,38^ proposed two versions of CDFT. In the first implementation,^37^ they combined CDFT with a frozen-density embedding approach. The method, termed constrained-subsystem DFT (CSDFT), is mainly applied to describe hole transfer reactions. In the later paper,^38^ they presented a CDFT method tailored to compute low-lying electronic excitations (XCDFT) of molecular systems, which resolves the space of virtual states by projection. The results show an accuracy only slightly worse than LR-TDDFT. A more recent paper by Roychoudhury *et al.*(39) proposes a generalization of CDFT for charge-compensating electronic excitations in molecules (XDFT). The obtained results are again comparable to TDDFT results.

Beyond the above methods, there are also other DFT-based approaches for excited-state calculations, which are either generally applicable, such as orthogonality-constrained DFT,^40^ or designed for CT states, such as constricted variational density functional theory.^41^ In short, there are many approaches which can compute low-lying excitations in molecular systems, with each displaying limitations either in the ability to describe particular classes of excited states (LE *vs.* CT) or in the maximum accessible system size.

Furthermore, beyond the ability to treat many atoms, the complexity of large systems often also necessitates the ability to map to local degrees of freedom (DoFs).^42,43^ To this end, it would be highly advantageous to have an excited-state method which can be related to a local description of a large system, for example, to excite a single molecule within a cluster of molecules. Given all these factors, there is no clear consensus on the best approach to use, particularly for applications such as TADF where the nature of the excitation can be a combination of LE and CT, and the effect of an extended environment can be crucial for accurately describing the excitation. Nonetheless, the recent variants of CDFT demonstrate its potential for providing accurate results with a lower computational cost than TDDFT.

In this paper, we present an alternative variation of CDFT. In our approach, which is implemented in the wavelet-based BigDFT code,^44^ the constraint is defined as a particular *transition*, or a combination of transitions, between given occupied and virtual states, rather than a region of the simulation space. The approach is therefore termed transition-based CDFT (T-CDFT). The transition constraint takes inspiration from an optical excitation term, rigorously defined using LR equations and parameterized, for instance, from LR-TDDFT. In this context, it can be considered as a further step beyond LR calculations, where SCF effects are added *on top* of the optical excitation. We consider both “pure” transitions between one occupied molecular orbital (the highest occupied molecular orbital) and a single orbital in the unoccupied sector—which require no additional simulation input—and “mixed” excitations involving more than one occupied → virtual transition, where we use TDDFT to define the transition breakdown. We benchmark our approach on low-lying singlet and triplet excitations of a set of molecules in the gas phase, including both acenes and OLED emitters, putting the results in comparison with ΔSCF and TDDFT calculations. Although the present work focuses on gas-phase simulations, we also describe a path toward future large-scale simulations, using the ability of BigDFT to treat systems containing thousands of atoms.^45,46^

The outline is as follows. In Section 2, we first present the underlying formalism of T-CDFT, before describing the implementation in BigDFT. We finish Section 2 by defining two indicators which will be used for analyzing excitations and specifying the Computational Details. In Section 3, we present the results, first discussing the nature of the excitations, including both LE/CT character and the effect of treating excitations as pure or mixed. We finish with a detailed comparison between the obtained excitation energies for the different methods, for both LE and CT excitations. Finally, in Section 4, we conclude.

## 2 Methods

### 2.1 Excitations in the Linear-Response Formalism

When a system is submitted to an excitation, its density matrix and therefore its observables are modified by the effect of a (potentially frequency-dependent) perturbing operator Φ̂(ω), with ω being the frequency. Stated otherwise, we can identify a response density operator ρ′^(ω) which represents the deviation of the density matrix from the ground-state equivalent, indicated by ρ̂_0_. Such a response density satisfies an equation of motion written in the form of a quantum Liouville (super) operator, , (see, e.g., ref (47)):
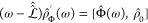
1

Its action on a generic operator *Ô* reads
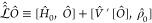
2where *Ĥ*_0_ is the ground-state Hamiltonian and *V′^*[*Ô*] ≡ ∫d**r**d**r**′δ*V̂*[ρ_0_*^*]/δρ(**r**, **r**′)*O*(**r**, **r**′) encodes the response of the ρ̂-dependent potential to a modification of the density operator. The “excitation modes” of the molecule are defined through the *excitation operatorsÊ*_a_, satisfying
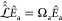
3with Ω_a_ being the excitation energies. The operator orthonormalization condition,
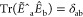
4where  is the excitation operator associated to the left Liouvillian eigenproblem,^48^ guarantees that the excitation operators can be seen as a basis for representing the perturbation of the system.

Under a linear-response condition, it is possible to show^48^ that the excitation operators satisfy the so-called *transverse* condition, which states that
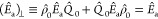
5where  is the projector to the empty subspace of the ground-state Hamiltonian *Ĥ*_0_. According to this condition, excitation operators can be parametrized as
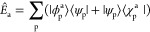
6

We here indicate ψ_*p*_ as the occupied orbitals, where ϕ_p_^a^ and χ_p_^a^ are associated to vectors belonging to the span of unoccupied states (and are therefore orthogonal to the set of all occupied orbitals).

Each excitation mode of the system, with associated energy Ω_a_, is thus described using a set of states {ϕ_p_^a^, χ_p_^a^}, each defined in the unoccupied subspace. It is possible to show^48^ that these objects represent, respectively, the state into which |ψ_*p*_⟩ is excited—or from which it decays—when the system is subject to the *monochromatic* perturbation Φ̂_a_ ≡ [*Ê*_a_, ρ̂_0_], which would only resonate with the excitation having an energy Ω_a_ (see^48^). The spectrum is symmetric with respect to the inversion of the eigenvalues Ω_a_ → −Ω_a_ and, given a specific excitation {ϕ_p_^a^, χ_p_^a^}, the associated solution with opposite energy is described using the transposed pair {χ_p_^a^, ϕ_p_^a^}.

### 2.2 Transition-Based Constrained DFT

Excited states, as calculated, for example, with LR-TDDFT, may be characterized by the orbitals involved in a given transition. Each of the occupied orbitals labeled ψ_p_ is then associated with a particular transition of this state in the unoccupied sector.

Following these guidelines, we define the (Hermitian) transition operator, *T̂*_a_
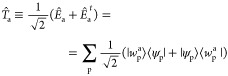
7which is associated with the linear combination of an excitation and a de-excitation with the same energy. Normalization of the excitation modes implies that ∑_p_⟨*w*_p_^a^|*w*_p_^a^⟩ = 1. A representation of the states |*w*_a_^p^⟩ ≡ |ϕ_p_^a^⟩ + |χ_p_^a^⟩ can be provided by introducing an explicit basis {|*s*⟩} in the subspace of empty states so that both |ϕ_p_^a^⟩ and ⟨χ_p_^a^| can be represented as
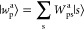
8

The representation of the above equation in the basis set of the eigenvalues of the ground-state Hamiltonian gives rise to the TDDFT Casida’s equations. For semi-local DFT functionals, the normalized coefficients *W*_ps_^a^ can be directly extracted from the Casida’s coupling matrix eigenproblem, thereby providing an explicit representation of |w_p_^a^⟩.

We define our excitation energies *via* the following equation
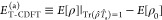
9where we denote *E*[ρ] as the SCF energy obtained from the density ρ. In other terms, we minimize the energy by imposing self-consistency under the transition constraint imposed by the operator *T̂*_a_. More specifically, the density matrix operator is constrained such as to include the transitions ψ_p_ → *w*_p_^a^ in its definition. The energy of the system is then minimized along the set of solutions implementing such a constraint.

#### 2.2.1 Transition Breakdown

We now describe the procedure for imposing the constraint on the density. A particular excitation (labeled a) is characterized by a set of occupied states |ψ_p_⟩, which are excited to the corresponding unoccupied orbitals |*s*⟩ with a weight provided by the coefficients *W*_ps_^a^. This may involve only one occupied p orbital, for example, the highest occupied molecular orbital (HOMO), or it may involve a mixture of several orbitals. We refer to the former as a “pure” transition, while the latter is considered a “mixed” transition. For a given orbital p, the level of purity of the transition a can be quantified by means of a transition purity indicator, , defined as
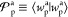
10which would lead to a value of 1 if only the orbital ψ_p_ participates in the transition. Clearly,  for any excitation a.

For the generic case of a mixed transition, it is then enough to split the T-CDFT approach into multiple constraints by decomposing the transition *T̂*_a_ into partial terms
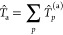
11where *T̂*_*p*_^(a)^ is a pure transition defined from the sole ket |*w*_*p*_^a^⟩; we can define the energy of the mixed transition by the SCF energy obtained from the density operator
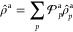
12where ρ̂_*p*_^a^ is the SCF density obtained from the pure T-CDFT calculation with the constraint Tr(ρ̂*T̂*_*p*_^(a)^) = 1.

#### 2.2.2 Extracting Singlet and Triplet Excitations

With this representation, the orbital sector of the configuration interaction space is, by construction, identical between up and down spins. Therefore, spin contamination is forbidden, and we work in the subspace of *S*_*z*_ = 0 excitations. Singlet (+) and triplet (−) transitions can be easily identified, taking the solutions with *W*_ps↑_^a^ = ±*W*_ps↓_^a^, respectively.

In the KS DFT formalism, ρ̂_0_ is found by SCF optimization of the following Lagrangian:
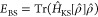
13where the KS Hamiltonian *H*_KS_ is functionally dependent on the density matrix. To impose the constraint, we may add to the above Lagrangian the following term
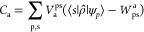
14The set of Lagrange multipliers  is there to enforce the condition
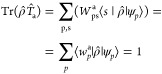
15which would add to the KS Hamiltonian, a density-independent term:
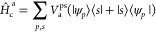
16where the sum should only be performed on the set of states *p*, *s* such that *W*_ps_^a^ ≠ 0. Once the appropriate values for the Lagrange multipliers are found, the energy of the excited system can be calculated with the usual KS expression, after removing the constraining term from *H*_KS_. Singlet and triplet transitions can then be associated with constraint operators, which are spin-averaged (+) and spin-opposite (−), respectively: (*Ĥ*_c_^a↑^ = ±*Ĥ*_c_^a↓^).

Such a formalism is particularly practical because for each pure transition *T̂*_*p*_^(a)^, one can choose an arbitrarily large Lagrange multiplier as the representability condition of the density matrix will always guarantee ⟨*w*_*p*_^a^|ρ̂|ψ_p_⟩ ≤ 1. A value of a magnitude of 20 atomic units for *V*_c_ is largely sufficient for imposing the constraint, as discussed in Section 2.5. Therefore, although the computational cost increases with the number of components in a given mixed excitation, the overall cost is comparable to traditional CDFT, where several calculations may be required to identify the Lagrange multiplier which satisfies the constraint.

### 2.3 Indicators for Analyzing Excitations

When analyzing excited states, it is useful to employ quantitative indicators, which allow the comparison of various features of a given excitation. This includes the orbitals involved in a given transition and the spatial character. To this end, we define the following two simple indicators.

#### 2.3.1 Transition Purity

Both the transition purity indicators,  (eq 10), and the T-CDFT formalism may be applied to any transition. In this work, a  refers to a pure HOMO-virtual excitation, while a number significantly below 1 indicates that excitations involving deeper occupied orbitals have a non-negligible contribution to the overall description of the excitation.

#### 2.3.2 Spatial Overlap

The accuracy of computed excitation energies strongly depends on whether the chosen method is able to correctly capture the character of the excitation. As discussed, this is particularly true for TDDFT, where energies of low-lying CT states calculated with some functionals may be severely underestimated, in some cases by several eV, while other functionals agree well with experiment.^49^ This has motivated the development of diagnostic tools for predicting the accuracy of TDDFT in a given case by classifying the nature of the excitation. It is not straightforward to develop a unique and effective descriptor, so that several examples of such developments can be found in the literature.^50−54^ These generally include geometric descriptors based on the molecular orbitals and electron densities.

One such descriptor is the Λ index,^54,55^ which is based on the overlap of molecular orbital moduli and is defined as
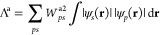
17

It has been suggested that a small orbital overlap, defined by a Λ value below 0.3–0.4, depending on the functional, corresponds to a CT transition which is not correctly described by TDDFT.^55^

Although it is possible to define indicators such as Λ which depend on the output of the excited state calculation, in this work, we instead employ a simplified descriptor which is based on a particular transition that is used as the constraint in T-CDFT, that is, the simulation input, rather than the output. For the case of pure HOMO–LUMO (lowest unoccupied molecular orbital) transitions, this results in a simplified version of the Λ index. This descriptor, which we denote Λ_T_, is based on the square root of the product of the overlap of solely the HOMO and LUMO wavefunctions and thus describes their *spatial overlap*
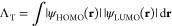
18

At the two extremes, a value of zero indicates no spatial overlap between the HOMO and LUMO and hence a CT excitation, while a value of 1 represents full spatial overlap, corresponding to a LE state. We note that Λ_T_ does not distinguish between singlet or triplet excitations, nor does it take into account additional contributions in the case of mixed excitations. For work requiring an in-depth analysis of a particular excitation, a modified version based on the output of T-CDFT or other indicators such as those referenced above would therefore be more appropriate.

### 2.4 Implementation in BigDFT

We have implemented T-CDFT in the BigDFT code,^44^ which uses a Daubechies wavelet basis.^56^ By taking advantage of the orthogonality, compact support, and smoothness of wavelets and in conjunction with accurate analytic pseudopotentials (PSPs), BigDFT is able to yield a high, systematically controllable precision. It has both a standard cubic scaling approach with respect to the number of atoms^57^ and a linear scaling (LS) algorithm, which can treat thousands of atoms.^45,46^ The T-CDFT implementation builds on the existing CDFT implementation in LS-BigDFT,^58,59^ wherein a support function (or wavefunction) basis is constructed for the ground state and then used as a fixed basis for the (T-)CDFT calculation. In the following, we therefore first summarize the support function (SF)-based approach employed in LS-BigDFT. We then describe the generation of both SFs and extended KS wavefunction basis sets, where the latter is used to verify the suitability of the SF basis for excited states.

#### 2.4.1 Linear Scaling BigDFT

In LS-BigDFT, the extended KS orbitals are expressed in terms of a set of localized SFs, ϕ_α_, *via* the coefficients *c*_*i*_^α^
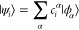
19The density matrix, ρ̂, is then defined in terms of the SFs and the density kernel, *K*^αβ^(60)
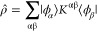
20

By taking advantage of the well-known nearsightedness principle,^60,61^ it is possible to impose strict localization on both the SFs and density kernels. In BigDFT, the SFs are represented in the underlying wavelet basis set and optimized *in situ* during the self-consistency cycle. This results in a set of localized SFs, which have adapted to their local chemical environment, giving a minimal basis which, by construction, can represent the occupied KS orbitals. Because the SFs are truncated within a user-defined localization radius, *R*_loc_, systematic convergence is possible by increasing the localization radius.

The density kernel is then also optimized, either by means of the Fermi operator expansion (FOE)^62,63^ approach, which works directly with the density kernel or with direct minimization or diagonalization approaches, which are used to obtain the coefficients *c*_*i*_^α^, from which the kernel can then be constructed. FOE used in conjunction with sparse matrix algebra, as implemented in the CheSS library,^42^ results in LS computational cost.

The localized SFs of LS-BigDFT can also be used as a means for further reducing the complexity of calculations of large systems. This is achieved *via* a fragment-based analysis, in which the system is divided into subsystems and can be used both to reduce the computational cost by exploiting similarity between fragments^58,64^ and to identify independent fragments and analyze interactions between them.^43,65^ SF-based T-CDFT is fully compatible with these fragment-based approaches; future work will aim at exploiting this to study excitations in environments.

#### 2.4.2 Support Function Basis

The use of a transition-based constraint relies on the ability to accurately represent both the occupied orbitals and the virtual orbital(s) involved in the constraint. The SFs are optimized to describe the occupied states; however, as in the ONETEP code,^66^ which uses a similar approach to optimize the basis of non-orthogonal generalized Wannier functions (NGWFs), the unoccupied states can be poorly represented.^67,68^ In ONETEP, this problem is overcome by using a projection operator to optimize a second set of NGWFs to represent the virtual states, which are then combined with the ground-state NGWFs.^68^

In LS-BigDFT, we instead retain a single set of SFs, exploiting the direct minimization approach to optimize selected virtual states alongside the occupied states.^45^ When only a few virtual states are required, this may be carried out in a single calculation. However, when a larger number of virtual states are required, it is more stable to employ a two-step approach:

1. Ground-state calculation for occupied states only, to obtain the density, and an initial guess for both the SFs and kernel.
2. Non-self-consistent calculation in which the SFs and kernel are further optimized to represent a number of virtual states.

Although step 1 may employ any approach to kernel optimization, step 2 requires the use of the direct minimization approach. Because the virtual states are more delocalized than the occupied states, particularly in the case unbound of states, it is typically necessary to use larger localization radii, while depending on the nature of the virtual states, it may also be necessary to increase the number of SFs.

#### 2.4.3 Wavefunction Basis

In order to validate T-CDFT with a SF basis, we have also employed a wavefunction (WFN)-based approach, wherein the basis set is generated *via* a ground-state cubic scaling calculation with a (large) number of virtual states. These wavefunctions are then used for T-CDFT, treating them as a fixed SF basis with effectively infinite localization radii. By increasing the number of virtual wavefunctions, it is possible to systematically approach the complete basis set limit, assuming that the set of excited states *w*_*p*_^a^ is localized (which is always true for excitations below a given threshold, see^48^). Such an approach is therefore possible for comparing the choice of the excitation operator in different computational setups.

### 2.5 Computational Details

Vertical singlet and triplet excitations were calculated using T-CDFT, ΔSCF, and TDDFT. T-CDFT calculations used PBE^69^ only, as hybrid functionals are not available in LS-BigDFT, while ΔSCF and TDDFT calculations used both PBE and PBE0.^70^ Because T-CDFT is targeted at large systems, where hybrid functionals are often prohibitively expensive, this reflects the computational setup which would be used on this scale. All ground-state calculations and singlet T-CDFT calculations are spin-restricted, while the remainder of the excited-state calculations are unrestricted.

ΔSCF energies and the spatial overlap parameter were calculated using cubic-scaling BigDFT, where excited-state calculations used the ground-state wavefunctions as an initial guess to avoid convergence on local minima, as described in the Supporting Information. The ΔSCF procedure notoriously models the non-Aufbau electronic singlet state which is not a spin eigenfunction.^71^ To obtain the energy of the singlet excited state, we therefore applied the common spin purification formula to the uncorrected mixed-state energy *E*_S1_^purified^
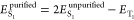
21All reported S_1_ ΔSCF energies are *E*_S1_^purified^.

TDDFT calculations employed NWChem^72^ using the Tamm-Dancoff approximation (TDA),^73^ with a cc-pVTZ basis set.^74^ LR-TDDFT calculations were also performed using BigDFT, using the full Casida formalism,^18^ in order to determine the transition breakdown and purity. As only LDA^75^ is available for TDDFT in BigDFT, these calculations, including the transition breakdowns, were performed using LDA with a WFN basis generated using PBE; the difference compared to using a basis generated with LDA was found to be negligible. T-CDFT calculations employed a SF basis with 4/9/9 SFs for H/C/N with *R*_loc_ = 4.23 Å; the calculated values were found to be within 0.05 eV of the converged WFN-based results, demonstrating that the SF basis is complete enough to allow accurate fixed-basis T-CDFT calculations, provided the virtual states of interest are well represented. A Lagrange multiplier of −20 was used for all T-CDFT calculations as this was found to be large enough to converge Tr(**KW**)—an example convergence plot is shown in the Supporting Information, alongside further Computational Details.

## 3 Results

**Figure 2:**
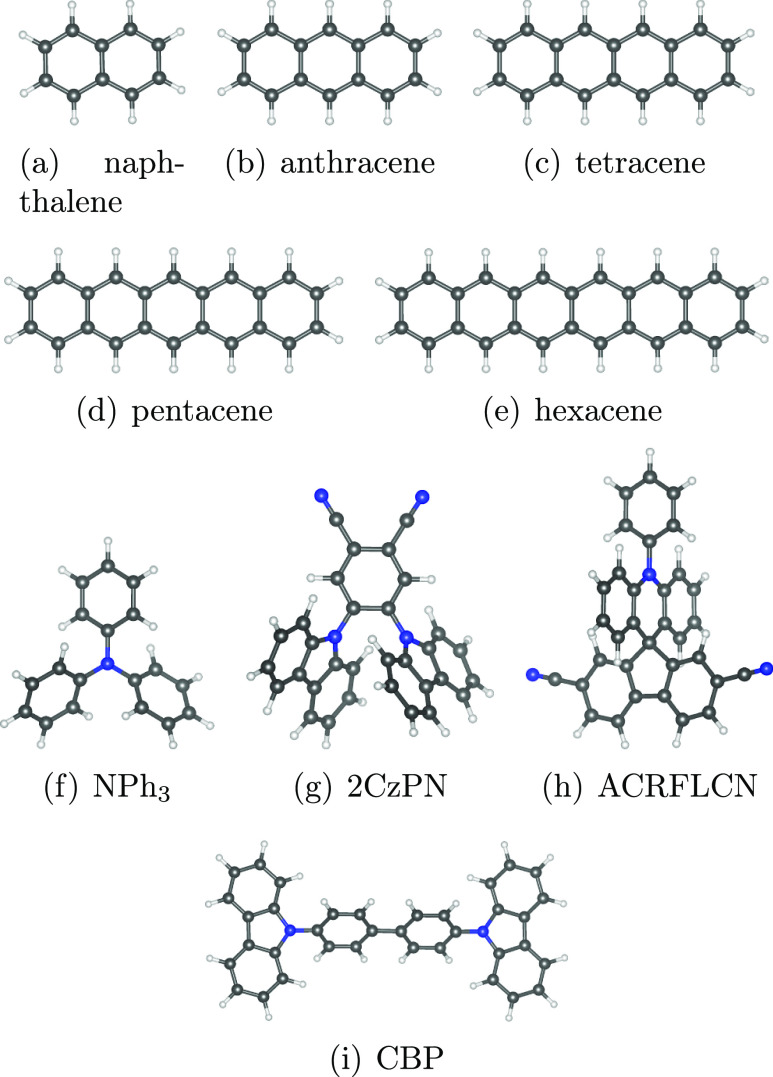
Relaxed atomic structures of the test set molecules. H/C/N atoms are depicted in white/black/blue, respectively.

Benchmark calculations of the lowest-energy singlet and triplet (*S*_1_ and *T*_1_) excitations were performed for a set of molecules which were chosen to provide a range of LE, CT, and mixed LE-CT character excitations. The test set, which is depicted in Figure 2, consists of five acenes, namely, naphthalene, anthracene, tetracene, pentacene, and hexacene, which constitute a set of well-characterized molecules and four exemplar OLED molecules, namely, NPh_3_ (triphenylamine), 2CzPN (1,2-*bis*(carbazol-9-yl)-4,5-dicyanobenzene), ACRFLCN (10-phenyl-10H-spiro(acridine-9,9- fluorene)-2,7-dicarbonitrile), and CBP (4,4′-*bis*(*N*-carbazolyl)-1,1′-biphenyl). NPh_3_, 2CzPN, and ACRFLCN are among the most investigated TADF emitters while CBP is a host molecule used to sensitize TADF emitters.^76^

Although experimental data for these systems are available, these are typically adiabatic excitation energies, which can differ significantly from vertical excitation energies (see, *e.g.*, ref (77)). Furthermore, such experiments take place under various external conditions, with the results being sensitive to the external environment (*i.e.*, the solvent or other molecular environment) and temperature.^78,79^ As the aim of this work is to assess the performance of T-CDFT for vertical excitations in the gas phase, it is therefore not informative to make quantitative comparisons with experimental data. Indeed, one of the motivations behind this work is to provide a formalism which can treat large enough systems to take into account explicit environmental effects. Such comparisons are therefore saved for future work, while in the following, we focus on theoretical comparisons only.

### 3.1 Nature of the Excitations

**Figure 3:**
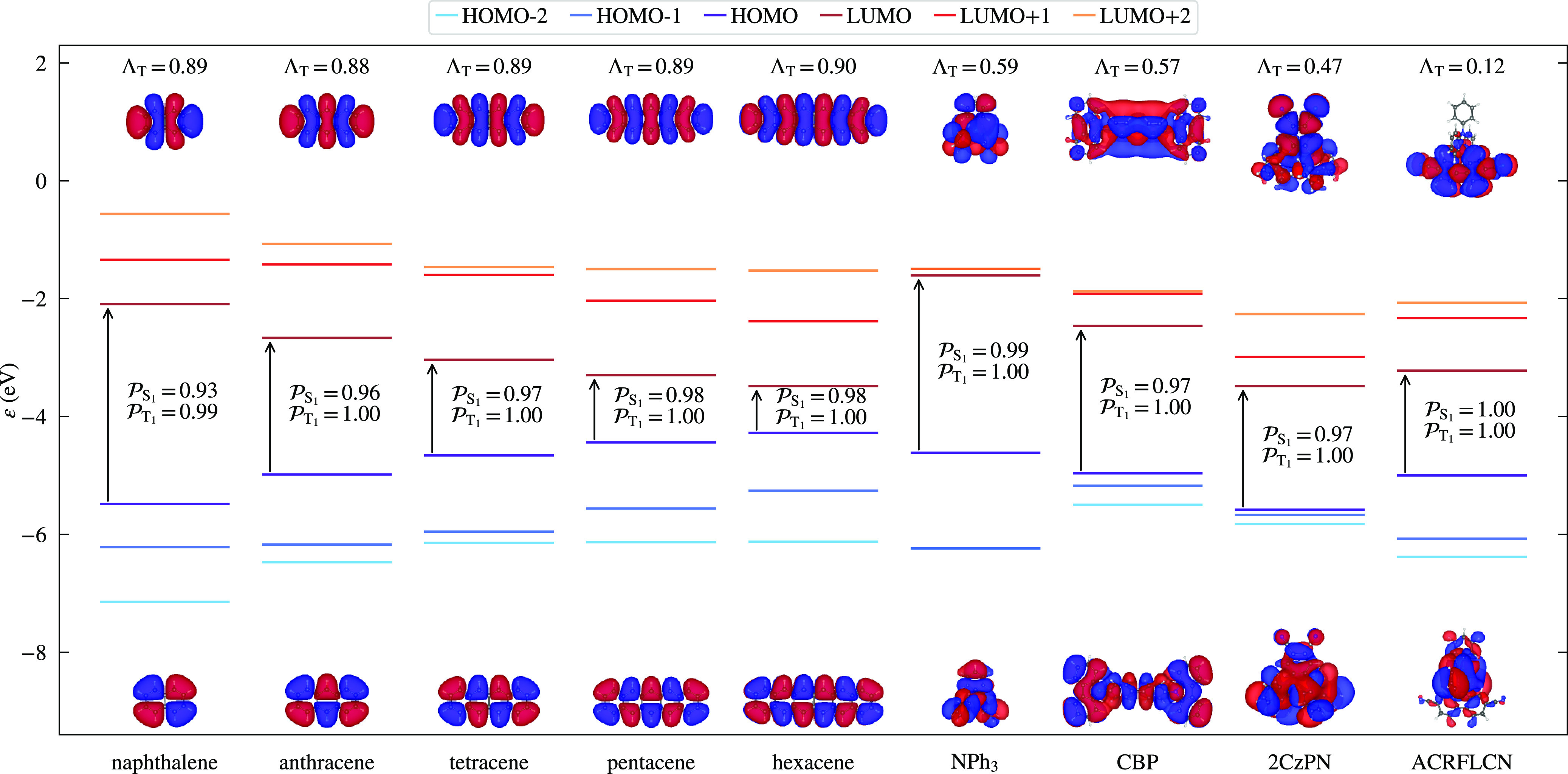
PBE-calculated frontier orbital energies and corresponding HOMO and LUMO wavefunctions, as obtained from cubic-scaling BigDFT. Wavefunctions were visualized in VESTA,^80^ using an isosurface value of 0.0005 *a*_0_^–3/2^. The corresponding charge transfer parameter, Λ_T_, and HOMO–LUMO transition purity values, , are also given for each molecule, where the latter are calculated using LDA with a PBE basis, as described in the text.

Before discussing the excitation energies, we first characterize the electronic excitations and component transitions for the benchmark molecules. The frontier orbitals for PBE are depicted in Figure 3, alongside  and Λ_T_ values. The equivalent PBE0 plot and the corresponding frontier orbital energies can be found in the Supporting Information.

#### 3.1.1 Transition Purity

The HOMO–LUMO transition purity values, , show that, within our computational setup, S_1_ excitations are less pure than T_1_ excitations, with the least pure excitation being 0.99 for T_1_ and 0.93 for S_1_. We therefore expect that a HOMO–LUMO constraint in T-CDFT should represent a reasonable approximation for these molecules; in order to test this, we treat the S_1_ excitations of the acenes as both pure and mixed. For the mixed excitations, the transition breakdown was taken from TDDFT, neglecting all contributions smaller than 0.01 and renormalizing the transition breakdown accordingly. The results are given in Table 1. For naphthalene, which has the least pure excitation (0.93 before normalization), the energy for the mixed excitation is around 0.1 eV higher than the pure case, whereas the mixed excitation energy for hexacene (0.98 purity before normalization) is only 0.02 eV higher than the pure excitation energy. For other excitations which are much less pure than naphthalene, the mixed nature of the excitations may have a much stronger effect on the calculated energies, although this will also will vary depending on the involved transitions and not just the relative contributions. For the purposes of this work, however, these results suggest that a purity of 0.97–0.98 or above is such that neglecting other contributions should make little difference to the results. Therefore, all OLED excitations are treated as pure, while the acene results in the following are those for the mixed excitations.

**Table 1:** Comparison of S_1_ Energies for the Acenes Calculated Using T-CDFT With PBE Both When Treated as Pure HOMO → LUMO Excitations and When Treated as Mixed Excitations Including all Transition Contributions Greater Than 0.01^a^

	HOMO → LUMO	HOMO-1 → LUMO+1	HOMO-2 → LUMO+2	energy
**naphthalene**				
pure	1.000			4.33
mixed	0.935	0.027	0.038	4.41
**anthracene**				
pure	1.000			3.09
mixed	0.975	0.010	0.015	3.15
**tetracene**				
pure	1.000			2.29
mixed	0.988	0.012		2.32
**pentacene**				
pure	1.000			1.75
mixed	0.989	0.011		1.77
**hexacene**				
pure	1.000			1.35
mixed	0.989	0.011		1.37

a Shown are the normalized transition contributions and the calculated energies in eV.

#### 3.1.2 Spatial Overlap

The high value of Λ_T_ for the acenes implies a strong spatial overlap between the HOMO and LUMO. Although this does not take into account the slightly mixed nature of the S_1_ excitations in the shorter acenes, as a first approximation it implies that the transition constraint is local in nature and will give rise to a predominantly local excitation (for both functionals). On the other hand, the OLED molecules have a smaller spatial overlap, so that the transition constraint and thus the excitation display a hybrid LE/CT nature of various degrees, in agreement with previous results.^9^ We therefore expect TDDFT with PBE to perform more robustly for the acenes, whereas the CT character found in the OLED excitations could lead to a less accurate description.

### 3.2 Acenes

**Figure 4:**
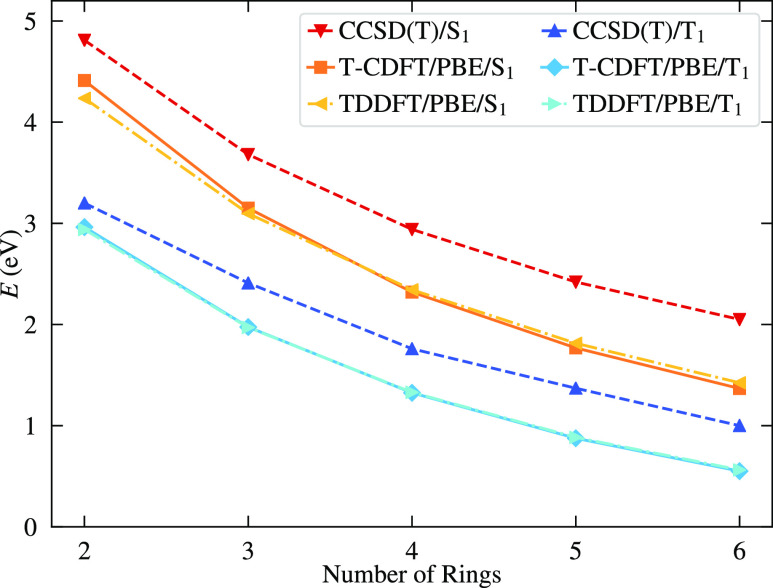
Trend in S_1_ and T_1_ energies for the acenes from naphthalene to hexacene, for both T-CDFT and TDDFT with PBE and CCSD(T), where the latter values are taken from ref (82).

We first consider the acenes, comparing our benchmark results with higher-level theory calculations based on CCSD(T), which is often regarded as the gold standard of chemical accuracy in quantum chemistry.^81^ We employ the values from ref (82) (and references within). The low-lying singlet excitations in the acenes are termed ^1^*L*_a_ and ^1^*L*_b_, which differ in energetic ordering depending on the acene in question.^82,83^ However, because the ^1^*L*_a_ state primarily involves a HOMO–LUMO transition and thus has the same character as our calculations, we take the ^1^*L*_a_ values as our reference, irrespective of whether the CCSD(T)-calculated ^1^*L*_b_ state is lower in energy. As shown in Figure 4, both T-CDFT and TDDFT with PBE capture the CCSD(T) trend in S_1_ and T_1_ energies, albeit with a systematic underestimation of both states, which increases slightly with the number of rings. This underestimation is common to all the DFT-based approaches (see Supporting Information for tabulated results).

**Figure 5:**
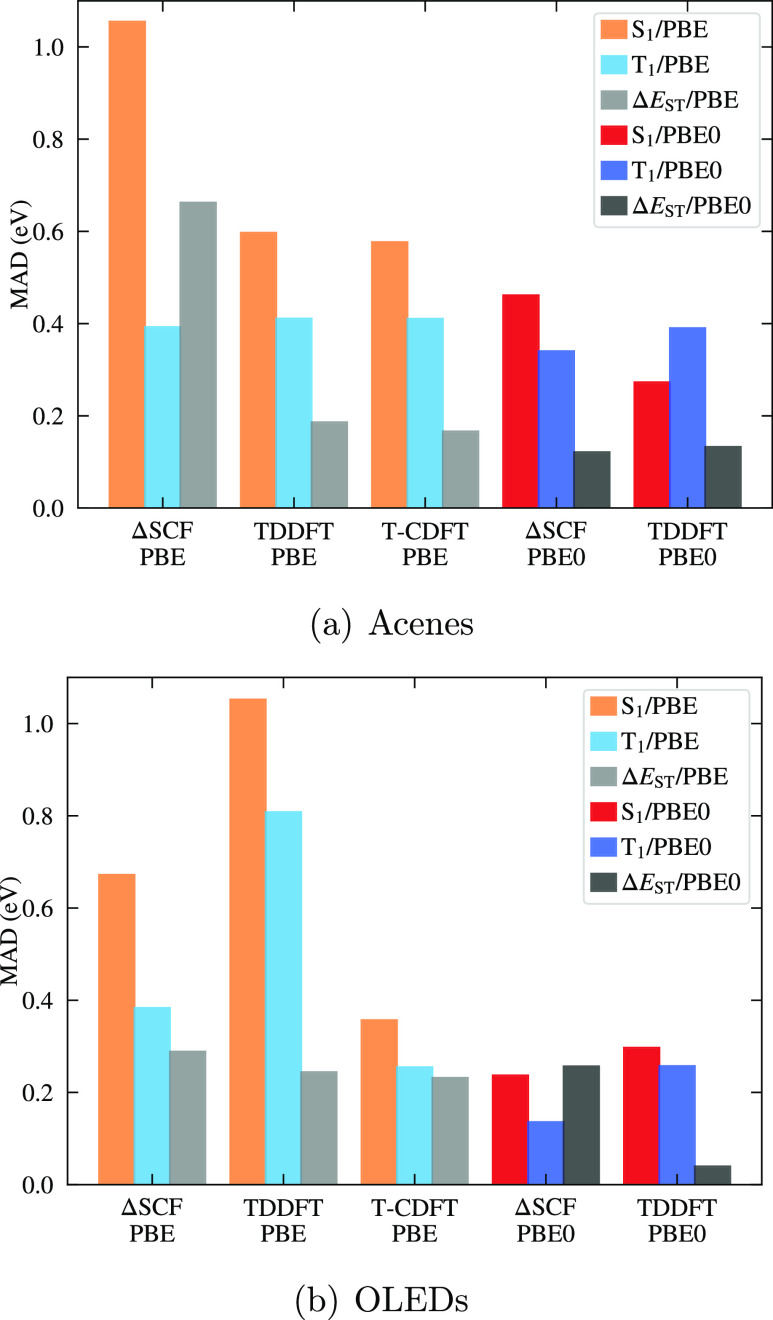
Mean absolute deviation (MAD) of benchmark vertical S_1_ and T_1_ energies for the set of molecules exhibiting pure excitations, relative to reference energies coming from CCSD(T)^82^ for the acenes and TDA-TDDFT with a tuned range-separated functional^84^ for the OLEDs. Corresponding energies are given in the Supporting Information.

Figure 5a shows the mean absolute deviation (MAD) between each of the DFT-based approaches and CCSD(T). Compared to T_1_, S_1_ is more sensitive to both the method and functional, with T_1_ energies being relatively consistent across the benchmark results and in most cases having a smaller MAD than S_1_. Furthermore, the T-CDFT/PBE results are in remarkable agreement with TDDFT/PBE results despite the lower computational cost, with both approaches having MADs of 0.6 and 0.4 eV for S_1_ and T_1_, respectively. Both T-CDFT and TDDFT significantly outperform ΔSCF for S_1_ when using PBE, while both ΔSCF and TDDFT with PBE0 give a modest improvement in accuracy, albeit at much higher computational cost. In short, we find that T-CDFT with PBE performs very well for the predominantly local excitations seen in the acenes.

### 3.3 OLEDs

Due to their larger size, there is a lack of higher-level quantum chemical reference data for the OLED molecules. However, due to the CT-like nature of the excitations, TDDFT with semi-local functionals cannot be expected to provide a reasonable reference. Indeed, the limitations of TDDFT for CT states are well known, as is the corresponding strong dependence on the employed functional. This has motivated the use of range-separated hybrid functionals for the treatment of TADF materials. For example, Adachi and co-workers^78^ reported that functionals such as CAM-B3LYP^85^ or LC-ωPBE^86^ tend to overestimate absorption energies for common TADF molecules. The situation may be improved by using “optimally” tuned range-separated functionals, which have been shown to give good agreement with experimental data,^84,87^ although the tuning of the separation parameters for a particular system increases the computational cost. Sun *et al.*(84) computed vertical excitation energies for a set of OLED molecules, including those considered in this work, using TDA-TDDFT with an optimally tuned LC-ωPBE* functional and a 6-31+G(d) basis set. We use these values as a reference in the following, although we note that they were performed using an implicit solvent (PCM toluene), which may influence the results.

**Figure 6:**
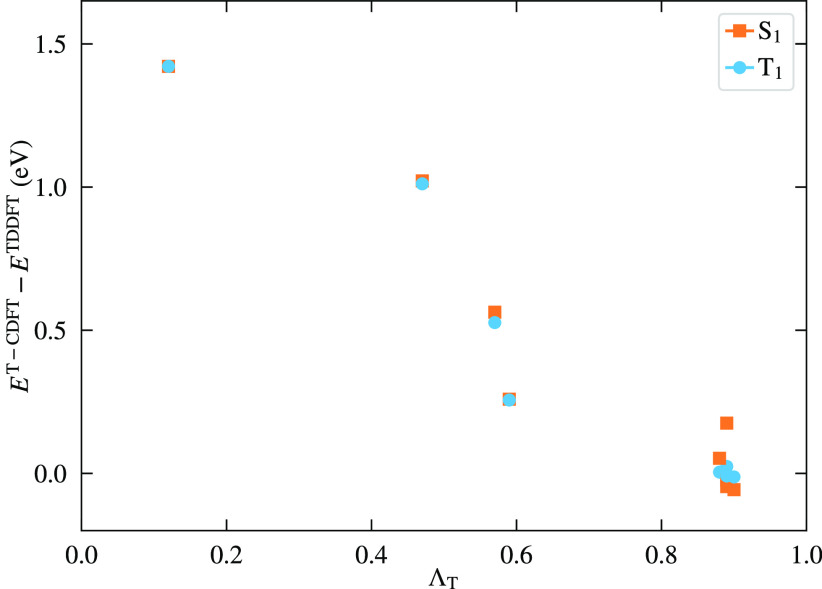
Difference between T-CDFT and TDDFT energies versus Λ_T_, the HOMO–LUMO spatial overlap, where both calculations employ the PBE functional.

The MAD between our calculations and the reference values is depicted in Figure 5b. There is a greater variability in MADs across methods and functionals compared to the acenes, particularly for T_1_. Furthermore, both ΔSCF and TDDFT with PBE systematically underestimate the reference values (see Supporting Information), with the large MAD for TDDFT/PBE being particularly striking. On the other hand, T-CDFT/PBE performs significantly better, giving MADs which are closer to the ΔSCF and TDDFT PBE0 values. This much better performance of T-CDFT/PBE compared to that of TDDFT/PBE is in line with the more CT-like nature of the excitations. Indeed, TDDFT/PBE most strongly underestimates the excitation energies for the molecules with the strongest CT-like character (*i.e.*, the smallest Λ_T_ values). At the same time, Figure 6 shows that the smaller the value of Λ_T_, the bigger the difference between T-CDFT/PBE and TDDFT/PBE. In other words, unlike TDDFT/PBE, which is strongly influenced by the nature of the transition, the quality of the T-CDFT/PBE results is not noticeably impacted by the nature of the excitation, giving reliable results for both the local excitations in acenes and the CT excitations in the OLED molecules.

Because accurate calculations of Δ*E*_ST_ are crucial for designing new, optimal TADF emitters, we conclude this section by discussing Δ*E*_ST_. Both ΔSCF and TDDFT with PBE benefit to some degree from error cancellations in S_1_ and T_1_ errors, so that there is less variability across the methods. Nonetheless, while T-CDFT with PBE underestimates the reference Δ*E*_ST_ values, this is less severe than the PBE-calculated ΔSCF and TDDFT values. Furthermore, the MAD for T-CDFT/PBE is similar to that of ΔSCF with PBE0 and only outperformed by the significantly more expensive TDDFT/PBE0 calculations.

To sum up, what emerges from our benchmark calculations and other computational works^9,88,89^ is that the modeling of the excitations in OLED molecules is strongly method- and functional-dependent, and it is therefore not trivial to obtain a unique and consistent description. Nonetheless, by comparing with tuned range-separated functional calculations, we see that, unlike TDDFT with PBE, T-CDFT is equally able to model both LE and CT states.

## 4 Conclusions

In this work, we introduce a variation of CDFT (T-CDFT), wherein the constraint is defined as a transition between particular occupied and virtual orbitals, rather than a region of the simulation space as in traditional CDFT. By defining an approach which goes beyond the linear response regime, we aim to provide a tool for the robust modeling of excitations in molecules. Our approach is applied to acenes and OLED emitters, for which the lowest-energy singlet and triplet states are dominated by a transition between the HOMO and LUMO. However, we also demonstrate the ability to take into account contributions from transitions between other orbitals. This has only a small impact on our benchmark calculations but could prove to be important in future investigations of excitations with a more strongly mixed character.

By comparing our benchmark calculations with reference values from the literature, we find that T-CDFT with PBE performs well for both the predominantly local excitations seen in the acenes and the mix of CT and LE character seen in the OLED emitters, outperforming or equaling both ΔSCF and TDDFT with the same functional. Importantly, T-CDFT does not suffer from the problems encountered when applying TDDFT with semi-local functionals to CT states and, unlike CDFT with a spatial constraint, can model both LE and CT states. At the same time, the computational cost of T-CDFT is similar to that of the ground state, while the ability to use a fixed (large) Lagrange multiplier keeps the cost significantly lower than TDDFT, even for mixed excitations involving multiple transitions. Furthermore, T-CDFT also proves to be more robust than ΔSCF, which can suffer from both spin contamination and convergence on local minima.

Finally, our approach has been implemented in the linear-scaling BigDFT code and is fully compatible with the available fragmentation approaches. This capability could be used to impose excitations on a per-fragment basis in supramolecular or large biological systems. For example, in the case of local excitations on a molecule (fragment) in a given environment, where no strong coupling with the environment is expected, the constraint could be imposed between orbitals associated with the target fragment only, while still treating the *full system*. Such an approach has the advantage of screening out spurious low-energy charge transfer excitations, which can be encountered with TDDFT. On the other hand, in the case where charge transfer excitations between fragments are of interest or where local excitations are expected to couple strongly with the environment, an alternative approach might be required. This could include performing TDDFT for a larger subset of the system or using other information about the excitations to guide the choice of constraint(s). Crucially, our framework is flexible enough to impose both intra- and inter-fragment constraints.

In summary, T-CDFT provides a robust and accurate approach for treating both LE and CT states. When combined with linear-scaling BigDFT, it is very well suited for treating excitations in large systems, enabling the exploration of explicit environmental effects on both excitation energies and Δ*E*_ST_, a key quantity for modeling TADF-based OLEDs. Indeed, we foresee that such an approach will represent a powerful tool for the study of excitations in realistic supramolecular morphologies, for applications such as TADF. Work in this direction is ongoing.

## 5 Data and Software Availability

In addition, the data associated with this work, including Jupyter notebooks and associated files which can be used to reproduce the calculations, are available at https://gitlab.com/martistella86/t-cdft-notebooks.

## Supporting Information Available

Additional computational details, discussion of the investigation into local minima in ΔSCF, example plot showing the effect of varying the Lagrange multiplier, plot of the frontier orbital energies as calculated with PBE0, tabulated data for the frontier orbital energies calculated using different basis sets, and both a plot and table containing the excitation energies calculated using the different approaches (PDF)
