# Supplementary material for: Transition-Based Constrained DFT for the Robust and Reliable Treatment of Excitations in Supramolecular Systems
Source: J Chem Theory Comput. 2022 Apr 26;18(5):3027–38. doi: 10.1021/acs.jctc.1c00548 (PMC9097287; doi:10.1021/acs.jctc.1c00548)
Supplement: Supplementary file 1 — ct1c00548_si_001.pdf [file ct1c00548_si_001.pdf]

Supplementary Information

Transition-Based Constrained DFT for the Robust and Reliable Treatment of Excitations in Supramolecular Systems

Martina Stella,[1,2] Kritam Thapa,[1] Luigi Genovese,[3] and Laura E. Ratcliff[1,4]

[1] *Department of Materials, Imperial College London, London SW7 2AZ, United Kingdom*
[2] *The Abdus Salam International Centre for Theoretical Physics, Condensed Matter and Statistical Physics, 34151 Trieste, Italy*
[3] *Univ. Grenoble Alpes, CEA, IRIG-MEM-L_Sim, 38000 Grenoble, France*
[4] *Centre for Computational Chemistry, School of Chemistry, University of Bristol, Bristol BS8 1TS, United Kingdom*

(Dated: April 25, 2022)

A. Additional Computational Details

BigDFT calculations were performed using HGH-GTH PSPs[1,2] with non-linear core corrections.[3] Gas phase geometry optimizations were performed for the ground state using PBE in BigDFT, with a maximum force threshold of 0.02 eV/Å. Single point calculations were performed with a wavelet grid spacing of 0.26 Å, while geometry optimizations employed a smaller grid spacing of 0.24 Å to ensure accurate force calculations. All BigDFT calculations used coarse and fine multipliers of 7 and 9 respectively. Cubic scaling BigDFT calculations employed a gradient convergence threshold of 10^{-5}. Due to the poor convergence behaviour of some NWChem calculations, a strict upper limit of 200 iterations was imposed for both ΔSCF and TDDFT calculations, beyond which calculations were considered not to have reached convergence.

T-CDFT WFN-based calculations were performed for a range of basis sizes, while SF-based calculations were performed for a range of localization radii for two basis set sizes – a minimal basis with 1/4/4 basis functions per H/C/N atom and a larger basis with 4/9/9 basis functions per H/C/N atom. The minimal basis was insufficient, giving energies almost 0.2 eV higher in energy than the more converged basis. On the other hand, increasing the localization radius from 4.23 to 5.29 Å led to differences less than 0.05 eV. We note that, due to the presence of additional degrees of freedom coming from environment molecules, it may be possible to use a smaller SF basis while retaining the same accuracy in future T-CDFT calculations of larger systems. All SF-based T-CDFT calculations were performed for a basis set optimized to represent all negative energy (bound) virtual states, as identified from the equivalent cubic scaling PBE calculation. No kernel truncation was applied, since the addition of the constraint decreases the locality of the kernel.

The HOMO-LUMO spatial overlap, Λ_{T}, was calculated as a post-processing calculation using the HOMO and LUMO wavefunctions extracted from cubic-scaling BigDFT. The HOMO-LUMO transition purity, $\mathcal{P}$, or the full transition breakdown in the case of mixed excitations, was also calculated as a post-processing step following BigDFT TDDFT calculations with LDA in a PBE-generated WFN basis, for which care was also taken to ensure the basis set contained enough virtual states to reach convergence. Although not used in this work, the functionality also exists to calculate transition purities using a SF basis. Jupyter notebooks and associated files for reproducing the BigDFT calculations are available at `https://gitlab.com/martistella86/t-cdft-notebooks`.

B. Local Minima in ΔSCF

For calculations which were performed using both BigDFT and NWChem, a close comparison of results was performed. In general, the results showed good agreement, however there were some large deviations between ΔSCF values between the two codes, which could not be attributed to basis set differences or the use of pseudopotentials, even when accounting for the fact that purification increases uncertainties in S_1 coming from basis set, PSP and convergence differences. Indeed, although generally considered a reliable tool for the computation of vertical HOMO-LUMO transitions in organic emitters,[4] because SCF algorithms are geared toward energy minimization, ΔSCF is known to sometimes collapse to low energy states. This can include the ground state,[5] although methods such as the maximum overlap method[6] have been developed to alleviate this problem. For some OLED molecules, it has also been observed to converge onto a different excited state due to a poor orbital guess or degeneracy.[7]

In this work no collapses onto the ground state were observed. However, large initial variations between BigDFT and NWChem energies were found, which could not be explained by basis set differences, even taking into account the fact that the use of the purification formula can exaggerate differences in singlet energies if the triplet states also differ. Instead, the variations were attributed to the presence of local minima, which was confirmed by inspecting the

charge density differences between the ground state and the corresponding excited state. It was found that using the ground state orbitals as an initial guess for excited state calculations eliminated these local minima, typically also improving convergence. This was confirmed by again inspecting the charge density differences.

C. Lagrange Multiplier

Fig. S1 shows the effect of varying the Lagrange multiplier for a pure excitation in naphthalene. A value of -20 was used in all calculations, giving $\mathrm{Tr}\,(\mathbf{KW})$ within 0.01 of $1e^{-}$ and thus confirming that there is no need to optimize V_c on a case-by-case basis.

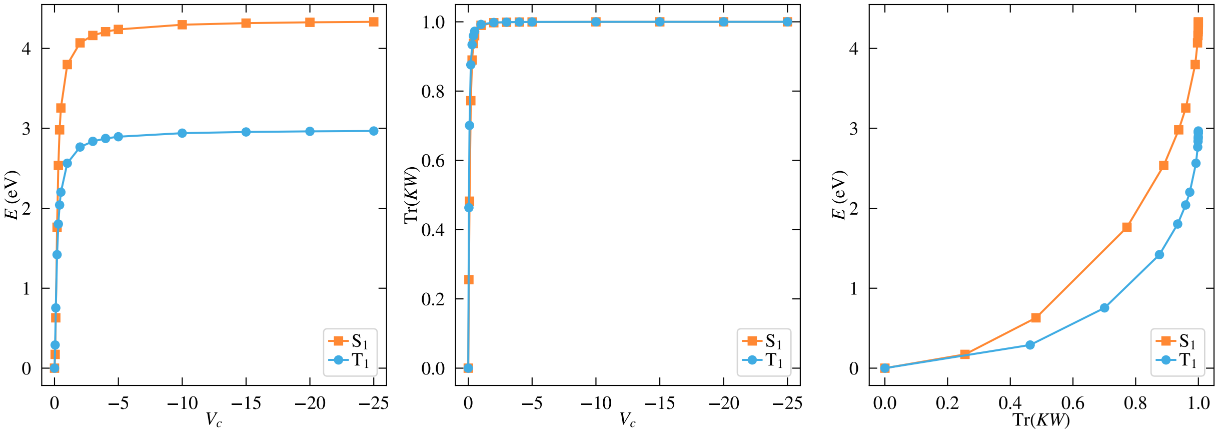

FIG. S1: Influence of the Lagrange multiplier, V_c, on the T-CDFT calculated singlet and triplet energies for a pure HOMO-LUMO constraint in naphthalene, as well as the constrained charge, $\mathrm{Tr}(KW)$, and the relation between the two. Calculations are performed for a SF basis with 4(9) SFs per H(C) atom, with localization radii $R_{\mathrm{loc}} = 4.23$ Å.

D. Additional Results

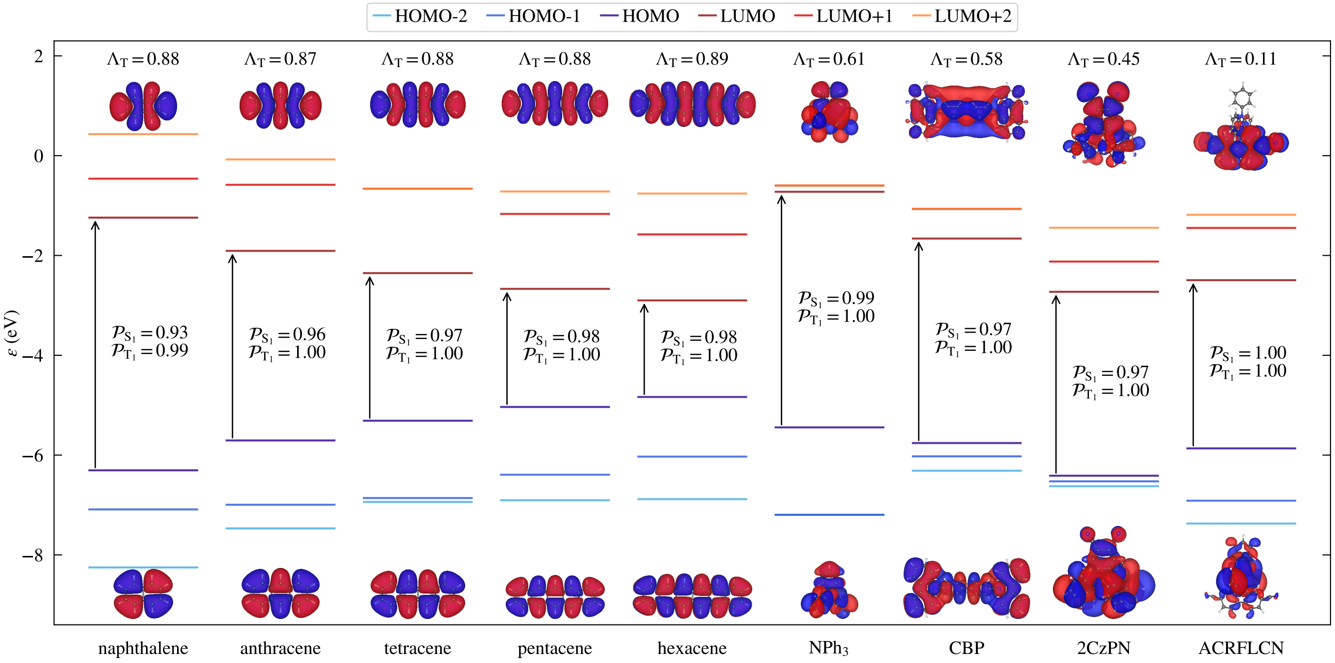

FIG. S2: PBE0-calculated frontier orbital energies and corresponding HOMO and LUMO wavefunctions, as obtained from NWChem using the cc-pVTZ basis. Wavefunctions were visualized in VESTA, using an isosurface value of 0.0005 $a_0^{-3/2}$. The corresponding charge transfer parameter, Λ_T and HOMO-LUMO transition purity values, $\mathcal{P}$, are also given for each molecule, where the latter are calculated using LDA with a PBE basis, as described in the main text. The corresponding energies are given in Tables S1 and S2.

TABLE S1: Frontier orbital energies and band gaps of the acenes, calculated using different functionals with the cc-pVTZ ('VTZ') basis set in NWChem, a wavelet basis using the cubic scaling approach of BigDFT and a SF basis of 4/9/9 SFs per H/C/N atom with $R_{\text{loc}} = 4.23$ Å. All values are in eV.

	PBE			PBE0	
	VTZ	wavelet	SF	VTZ	wavelet
**naphthalene**					
HOMO-2	-7.11	-7.15	-7.16	-8.23	-8.25
HOMO-1	-6.18	-6.22	-6.23	-7.07	-7.09
HOMO	-5.45	-5.48	-5.50	-6.30	-6.30
LUMO	-2.04	-2.09	-2.09	-1.22	-1.24
LUMO+1	-1.28	-1.34	-1.30	-0.43	-0.46
LUMO+2	-0.43	-0.56	-0.45	0.59	0.43
gap	3.40	3.39	3.41	5.07	5.06
**anthracene**					
HOMO-2	-6.44	-6.47	-6.48	-7.46	-7.47
HOMO-1	-6.14	-6.17	-6.18	-6.98	-6.99
HOMO	-4.95	-4.98	-5.00	-5.70	-5.71
LUMO	-2.63	-2.67	-2.67	-1.90	-1.91
LUMO+1	-1.37	-1.42	-1.38	-0.57	-0.58
LUMO+2	-1.00	-1.07	-1.03	-0.03	-0.08
gap	2.32	2.32	2.33	3.80	3.80
**tetracene**					
HOMO-2	-6.11	-6.15	-6.16	-6.93	-6.94
HOMO-1	-5.92	-5.95	-5.97	-6.86	-6.86
HOMO	-4.63	-4.66	-4.67	-5.31	-5.31
LUMO	-3.00	-3.04	-3.05	-2.35	-2.35
LUMO+1	-1.55	-1.60	-1.59	-0.65	-0.66
LUMO+2	-1.42	-1.46	-1.46	-0.65	-0.66
gap	1.62	1.62	1.62	2.96	2.96
**pentacene**					
HOMO-2	-6.10	-6.13	-6.15	-6.90	-6.90
HOMO-1	-5.53	-5.56	-5.58	-6.39	-6.39
HOMO	-4.41	-4.44	-4.45	-5.04	-5.04
LUMO	-3.27	-3.30	-3.31	-2.67	-2.67
LUMO+1	-2.00	-2.03	-2.04	-1.17	-1.17
LUMO+2	-1.46	-1.50	-1.49	-0.72	-0.72
gap	1.14	1.14	1.14	2.37	2.37
**hexacene**					
HOMO-2	-6.10	-6.12	-6.14	-6.88	-6.88
HOMO-1	-5.23	-5.26	-5.27	-6.03	-6.03
HOMO	-4.25	-4.28	-4.29	-4.85	-4.84
LUMO	-3.46	-3.48	-3.49	-2.90	-2.90
LUMO+1	-2.35	-2.38	-2.39	-1.58	-1.58
LUMO+2	-1.49	-1.52	-1.51	-0.76	-0.76
gap	0.79	0.80	0.80	1.94	1.93

TABLE S2: Frontier orbital energies and band gaps of the OLED molecules, calculated using different functionals with the cc-pVTZ (‘VTZ’) basis set in NWChem, a wavelet basis using the cubic scaling approach of BigDFT and a SF basis of 4/9/9 SFs per H/C/N atom with $R_{\mathrm{loc}} = 4.23$ Å. All values are in eV.

	PBE			PBE0	
	VTZ	wavelet	SF	VTZ	wavelet
**NPh3**					
HOMO-2	-6.20	-6.24	-6.28	-7.18	-7.20
HOMO-1	-6.20	-6.24	-6.28	-7.18	-7.20
HOMO	-4.57	-4.61	-4.65	-5.43	-5.45
LUMO	-1.53	-1.60	-1.60	-0.67	-0.72
LUMO+1	-1.43	-1.50	-1.51	-0.55	-0.60
LUMO+2	-1.43	-1.49	-1.51	-0.55	-0.60
gap	3.04	3.01	3.05	4.77	4.72
**CBP**					
HOMO-2	-5.46	-5.50	-5.52	-6.31	-6.31
HOMO-1	-5.14	-5.17	-5.20	-6.01	-6.03
HOMO	-4.92	-4.96	-4.99	-5.75	-5.76
LUMO	-2.41	-2.46	-2.47	-1.65	-1.66
LUMO+1	-1.86	-1.92	-1.91	-1.04	-1.07
LUMO+2	-1.82	-1.88	-1.88	-1.04	-1.07
gap	2.51	2.50	2.52	4.11	4.10
**2CzPN**					
HOMO-2	-5.78	-5.82	-5.85	-6.59	-6.62
HOMO-1	-5.63	-5.67	-5.70	-6.49	-6.53
HOMO	-5.54	-5.58	-5.61	-6.38	-6.41
LUMO	-3.43	-3.48	-3.50	-2.70	-2.73
LUMO+1	-2.93	-2.99	-3.01	-2.08	-2.12
LUMO+2	-2.20	-2.26	-2.27	-1.39	-1.44
gap	2.11	2.10	2.11	3.68	3.69
**ACRFLCN**					
HOMO-2	-6.34	-6.38	-6.40	-7.35	-7.37
HOMO-1	-6.03	-6.07	-6.09	-6.90	-6.91
HOMO	-4.95	-5.00	-5.02	-5.85	-5.87
LUMO	-3.17	-3.22	-3.23	-2.48	-2.49
LUMO+1	-2.26	-2.33	-2.32	-1.40	-1.45
LUMO+2	-2.01	-2.07	-2.07	-1.16	-1.19
gap	1.79	1.78	1.79	3.37	3.37

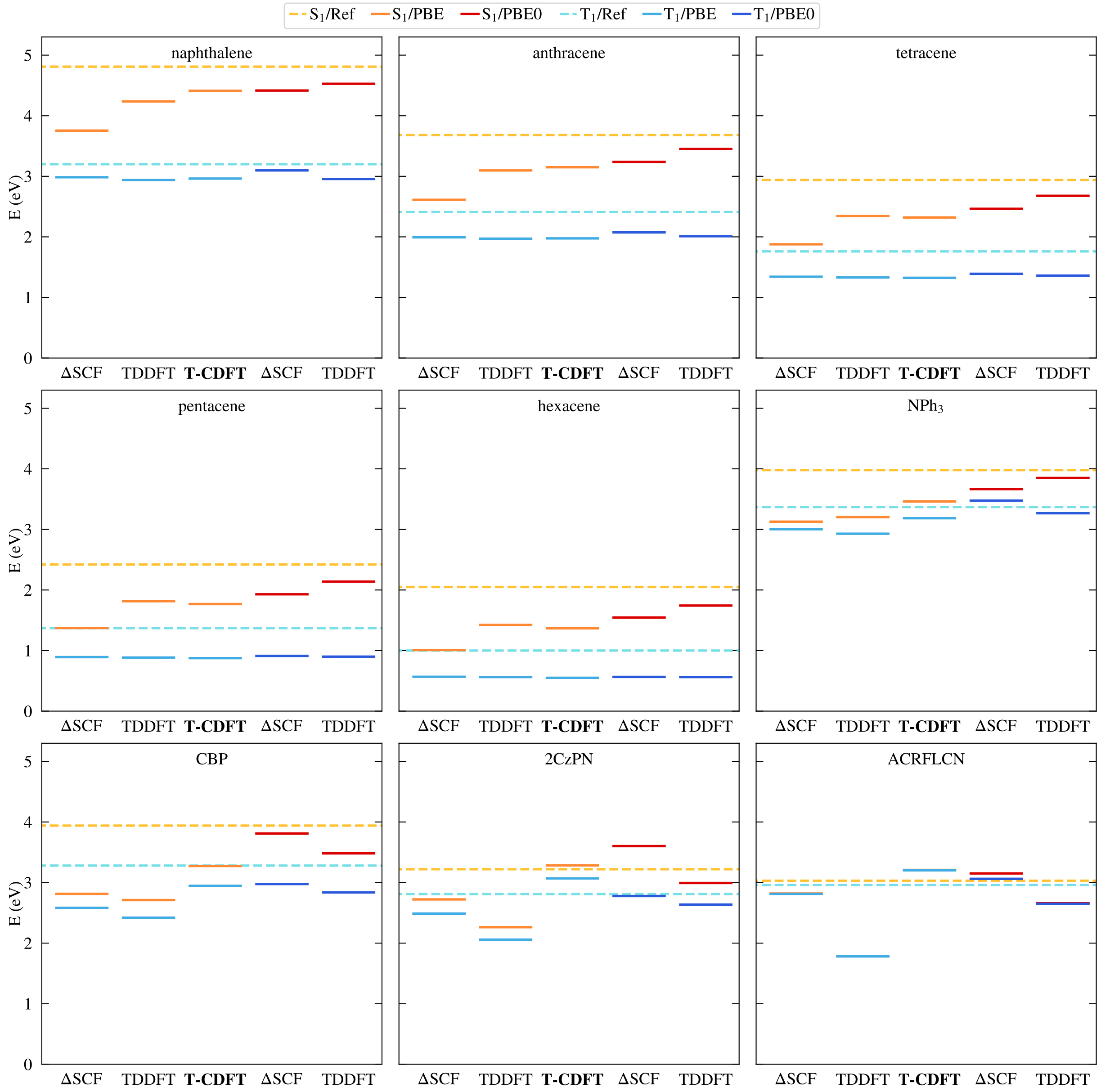

FIG. S3: Vertical S_1 and T_1 energies computed using different methods (T-CDFT, ΔSCF, TDDFT) and functionals (PBE, PBE0) using BigDFT and NWChem. Corresponding energies are given in Table S3, while the reference energies are calculated using CCSD(T)[8,9] for the acenes, and TDA-TDDFT with a tuned range-separated functional[10] for the OLEDs.

TABLE S3: Vertical S_1 and T_1 energies and singlet-triplet splittings, ΔE_{ST}, in eV, calculated using different methods (T-CDFT, ΔSCF, TDDFT) and functionals (PBE, PBE0) using BigDFT and NWChem, as compared to reference values from CCSD(T)[8,9] for the acenes, and TDA-TDDFT with a tuned range-separated functional[10] for the OLEDs. Also given is the HOMO-LUMO transition purity, $\mathcal{P}$, the spatial overlap, Λ_T, and the mean absolute deviation (MAD) of the different quantities with respect to the reference values.

			PBE				PBE0		
	Ref	$\mathcal{P}$	Λ_{T}	ΔSCF	TDDFT	T-CDFT	Λ_{T}	ΔSCF	TDDFT
**naphthalene**									
S_1	4.81	0.93		3.75	4.24	4.41		4.42	4.53
T_1	3.20	0.99	0.89	2.98	2.94	2.96	0.88	3.10	2.96
ΔE_{ST}	1.61			0.77	1.30	1.36		1.32	1.57
**anthracene**									
S_1	3.68	0.96		2.61	3.10	3.15		3.24	3.45
T_1	2.41	1.00	0.88	1.99	1.97	1.97	0.87	2.07	2.01
ΔE_{ST}	1.27			0.62	1.13	1.12		1.16	1.44
**tetracene**									
S_1	2.94	0.97		1.88	2.34	2.32		2.46	2.68
T_1	1.76	1.00	0.89	1.34	1.33	1.32	0.88	1.39	1.36
ΔE_{ST}	1.18			0.54	1.01	0.97		1.07	1.32
**pentacene**									
S_1	2.42	0.98		1.37	1.81	1.75		1.93	2.14
T_1	1.37	1.00	0.89	0.89	0.88	0.88	0.88	0.91	0.90
ΔE_{ST}	1.05			0.48	0.93	0.87		1.02	1.24
**hexacene**									
S_1	2.05	0.98		1.01	1.42	1.37		1.55	1.74
T_1	1.00	1.00	0.90	0.57	0.56	0.55	0.89	0.56	0.56
ΔE_{ST}	1.05			0.44	0.86	0.80		0.98	1.18
**MAD**									
S_1				1.06	0.60	0.58		0.46	0.27
T_1				0.39	0.41	0.41		0.34	0.39
ΔE_{ST}				0.66	0.19	0.17		0.12	0.13
**NPh_3**									
S_1	3.98	0.99		3.13	3.20	3.46		3.67	3.85
T_1	3.37	1.00	0.59	3.00	2.93	3.19	0.61	3.47	3.27
ΔE_{ST}	0.61			0.13	0.27	0.28		0.19	0.58
**CBP**									
S_1	3.94	0.97		2.81	2.71	3.27		3.81	3.48
T_1	3.28	1.00	0.57	2.58	2.42	2.95	0.58	2.98	2.84
ΔE_{ST}	0.66			0.23	0.29	0.33		0.83	0.64
**2CzPN**									
S_1	3.22	0.97		2.72	2.26	3.28		3.60	2.99
T_1	2.81	1.00	0.47	2.49	2.06	3.07	0.45	2.78	2.64
ΔE_{ST}	0.41			0.23	0.20	0.21		0.82	0.36
**ACRFLCN**									
S_1	3.03	1.00		2.82	1.79	3.21		3.15	2.66
T_1	2.96	1.00	0.12	2.81	1.78	3.20	0.11	3.06	2.65
ΔE_{ST}	0.07			0.01	0.01	0.01		0.09	0.01
**MAD**									
S_1				0.67	1.05	0.36		0.24	0.30
T_1				0.38	0.81	0.25		0.14	0.26
ΔE_{ST}				0.29	0.24	0.23		0.26	0.04

[1] S. Goedecker, M. Teter, and J. Hutter, Phys. Rev. B **54**, 1703 (1996).

[2] C. Hartwigsen, S. Goedecker, and J. Hutter, Phys. Rev. B **58**, 3641 (1998).

[3] A. Willand, Y. O. Kvashnin, L. Genovese, A. Vázquez-Mayagoitia, A. K. Deb, A. Sadeghi, T. Deutsch, and S. Goedecker, J. Chem. Phys. **138**, 104109 (2013).

[4] T. Kowalczyk, S. R. Yost, and T. V. Voorhis, J. Chem. Phys **134**, 054128 (2011).

[5] S. Bourne Worster, O. Feighan, and F. R. Manby, J. Chem. Phys. **154**, 124106 (2021).

[6] A. T. B. Gilbert, N. A. Besley, and P. M. W. Gill, J. Phys. Chem. A **112**, 13164 (2008).

[7] K. Zhao, Ö. H. Omar, T. Nematiaram, D. Padula, and A. Troisi, J. Mater. Chem. C **9**, 3324 (2021).

[8] T. Rangel, S. M. Hamed, F. Bruneval, and J. B. Neaton, J. Chem. Phys **146**, 194108 (2017).

[9] K. Lopata, R. Reslan, M. Kowalska, D. Neuhauser, N. Govind, and K. Kowalski, J. Chem. Theory Comput. **7**, 3686 (2011).

[10] H. Sun, C. Zhong, and J.-L. Brédas, J. Chem. Theory Comput. **11**, 3851 (2015).
